# MED16/383: How to Feed a System for Internet-based Continuing Medical Education for General Practitioners with Relevant Contents?

**DOI:** 10.2196/jmir.1.suppl1.e59

**Published:** 1999-09-19

**Authors:** C Dickmann, E Habermeyer, K Spitzer

**Affiliations:** ^1^Institut für Medizinische Informatik der RWTH Aachen, AachenGermany

**Keywords:** Internet, Continuing Medical Education, Computer Literacy, Multimedia, General Practitioner

## Abstract

**Introduction:**

Traditional Continuing Medical Education (CME) often means costly and inefficient learning for General Practitioners (GPs). The PostDoc project funded by the European Union aims at setting up and testing a model for a WWW-based learning environment that efficiently provides GPs with information, knowledge and skills. Results of the German project partners in setting up models and mechanisms to enable GPs to also work as content providers in their own information service are reported.

**Methods:**

Content and functionality of the Web-based learning environment is developed by participatory design and prototyping in collaboration of Psychiatrists, computer scientists and a volunteer user group of 10 GPs. Several questionnaires and user meetings have been performed. Standard WWW technology is used. An evaluation with 30-50 GPs will be started in July 1999.

**Results:**

The German service includes a multimedia training application for diagnosing psychiatric disorders, selected existing WWW information sources, indexes, and communication facilities. A calendar of CME events, medical news, and information on colleagues and healthcare regions is generated from databases that can be edited by WWW-based tools. The 10 GPs, practising 19 years on average, are mostly inexperienced in internet usage. They are willing to work with the service 37 minutes per week (15-60 min/w). 80% prefer a participatory-based design of the service. 50% desire a self-organized service. All GPs highly value the integrated information and learning environment but most tend to reject communication, collaboration or active editorial work. We set up a model that enables a GP to control his own level of participation in the service, avoiding bureaucratic impediments. GPs have one of these three roles: Unregistered visitors merely obtain public information, have restricted access and may send email feedback or requests. Registered users have a password that lets them access the whole functionality of the service. Editors have a password that also permits them to enter data directly in the databases. Each GP of the user group worked with the prototype a few hours in total. None of them participates as an editor in the service. Despite hands-on workshops and positive attitudes, it does not seem trivial to motivate GPs to become active in the WWW-based learning and information service.

**Discussion:**

Traditionally oriented GPs in our user group had difficulties in expressing their requirements to a Web-based CME environment. Co-operation was not as easy as expected before. The main reasons for that are massive time constraints, a lack of experience with the internet, GPs' individualism, and the habit to use traditional ways of CME. Active content provision by GPs seems only realistic if it is easy and can be done quickly. GPs should control the quality of the service's contents. During the Congress we will present detailed evaluations from 30-50 GPs working with the user roles.

